# Advancing HER2-low breast cancer management: enhancing diagnosis and treatment strategies

**DOI:** 10.2478/raon-2024-0030

**Published:** 2024-06-11

**Authors:** Simona Borstnar, Ivana Bozovic-Spasojevic, Ana Cvetanovic, Natalija Dedic Plavetic, Assia Konsoulova, Erika Matos, Lazar Popovic, Savelina Popovska, Snjezana Tomic, Eduard Vrdoljak

**Affiliations:** Institute of Oncology Ljubljana, Slovenia; Institute for Oncology and Radiology of Serbia, Medical Faculty, University of Belgrade, Serbia; Department of Oncology, Medical Faculty University of Niš; Clinic of Oncology, University Clinical Centre Niš, Serbia; University Hospital Centre Zagreb, School of Medicine, University of Zagreb, Croatia; National Cancer Hospital, Sofia, Bulgaria; Oncology Institute of Vojvodina, Faculty of Medicine, University Novi Sad, Novi Sad, Serbia; Medical University Pleven, Bulgaria; University Hospital of Split, University of Split - School of Medicine, Croatia

**Keywords:** HER2-low, metastatic breast cancer, Balkans, testing, innovative treatment, access

## Abstract

**Background:**

Recent evidence brought by novel anti-human epidermal growth factor receptor 2 (HER2) antibody-drug conjugates is leading to significant changes in HER2-negative breast cancer (BC) best practices. A new targetable category termed ‘HER2-low’ has been identified in tumors previously classified as ‘HER2-negative’. Daily practice in pathology and medical oncology is expected to align to current recommendations, but patient access to novel anticancer drugs across geographies might be impeded due to local challenges.

**Materials and methods:**

An expert meeting involving ten regional pathology and oncology opinion leaders experienced in BC management in four Central and Eastern Europe (CEE) countries (Bulgaria, Croatia, Serbia, Slovenia) was held. Herein we summarized the current situation of HER2-low metastatic BC (mBC), local challenges, and action plans to prevent delays in patient access to testing and treatment based on expert opinion.

**Results:**

Gaps and differences at multiple levels were identified across the four countries. These included variability in the local HER2-low epidemiology data, certification of pathology laboratories and quality control, and reimbursement conditions of testing and anticancer drugs for HER2-negative mBC. While clinical decisions were aligned to international guidelines in use, optimal access to testing and innovative treatment was restricted due to significant delays in reimbursement or limitative reimbursement conditions.

**Conclusions:**

Preventing delays in HER2-low mBC patient access to diagnosis and novel treatments is crucial to optimize outcomes. Multidisciplinary joint efforts and pro-active discussions between clinicians and decision makers are needed to improve care of HER2-low mBC patients in CEE countries.

## Introduction

In the era of precision medicine, the diagnostic and treatment landscape in oncology has progressively become more biomarker driven.^1,2^ In solid tumors, an early example of biomarkers with predictive value was the human epidermal growth factor receptor 2 (HER2), with positive results predicting response to targeted treatment with anti-HER2 monoclonal antibodies but no benefit for HER2-negative tumors.^1^ In breast cancer (BC), HER2 overexpression/gene amplification determined by immunohistochemistry (IHC) and/or in situ hybridization (ISH) is found in 15−20% of all tumors.^3,4,5^ Anti-HER2-directed therapies have significantly improved the survival of patients with both early and metastatic HER2-positive BC and consequently changed the treatment paradigm, being accepted as the standard of care throughout the world.^5,6^ The current pathology guidelines define HER2-positive tumors when the IHC score is 3+ or 2+ with the HER2 encoding gene (erb-b2 receptor tyrosine kinase 2 [*ERRB2*]) amplification by ISH (ISH-positive), whereas HER2-negative tumors have IHC scores of 0+, 1+, or 2+/ISH-negative.^7^

In light of recent evidence brought by novel anti-HER2 antibody-drug conjugates (ADCs)^5,8,9,10,11,12^, the current knowledge of HER2 expression range and its clinical applicability is changing since a significant proportion of HER2-negative tumors are in fact characterized by a spectrum of HER2 expression levels.^13,14^ A new targetable category has been identified in patients whose tumors are scored IHC 1+ or 2+/ISH-negative^3^, and this low level of HER2 expression has been termed ‘HER2-low’.^15^ HER2-low status is detected in 45−55% of all BC tumors: around two-thirds (65%) in hormone receptor-positive (HR+) BC and one-third (36%) in HR-negative (HR-) cancers.^6,16^ Treatment paradigms for both HR+ and HR-BC with HER2-low expression are evolving at a fast pace, leading to a new ‘revolution’.^17^ The clinical trial DESTINY-Breast04 (DB-04), evaluating trastuzumab deruxtecan (T-DXd) in patients with HER2-low advanced BC previously treated with chemotherapy, showed significant and clinically meaningful progression-free survival (PFS) and overall survival (OS) improvements and a manageable safety profile as compared with conventional chemotherapy (PFS 10.1 months for T-DXd vs 5.4 months in the physician’s choice of chemotherapy, hazard ratio=0.64, P=0.003, and OS 23.9 months for T-DXd vs 16.8 months in the physician’s choice of chemotherapy, hazard ratio=0.64, P=0.001, respectively).^12^ These results, together with the other ADC data, demonstrate the clinical relevance of HER2-low expression and are transforming the current understanding, and therefore management, of HER2-negative BC.^5,8,9,10,11,12,17,18,19,20^

Despite guideline recommendations for HER2-low diagnosis and European Society for Medical Oncology (ESMO) consensus reached for its treatment^5,7^, implementing guidelines in a real-life setting is a lengthy and difficult process, partly due to the diverse accessibility of novel anticancer medicines. In countries in Central and Eastern Europe (CEE), significant delays in patient access to innovative oncology treatments have been previously described.^21,22,23,24^ In an attempt to avoid such delays with ADCs in HER2-low BC, an expert meeting was held to identify the challenges and local unmet needs, and to find solutions to optimize access to diagnostics and adequate treatment of HER2-low metastatic BC (mBC) for patients from CEE. In this paper, we discuss the current situation pertaining to the overall diagnosis and management of HER2-low mBC and propose potential solutions to address the unmet needs in four CEE countries; we also consider similar situations, and solutions that may apply to many other former or current transitional countries throughout the world.

## Methods

A panel of ten opinion leaders was organized as part of a virtual meeting logistically supported by AstraZeneca and held on June 12, 2023. Eight medical oncologists and two pathologists from academic centers and/or national institutes of oncology in Bulgaria, Croatia, Serbia, and Slovenia with experience in the diagnosis, management, and follow-up of mBC patients from the CEE region were individually approached and further agreed to participate in the panel discussion. A pre-meeting survey was developed specifically for this project and reviewed by experts. The experts responded in anonymized manner to the preliminary survey, which included 31 questions grouped in the following four topics: epidemiology, biology, pathologic diagnosis, and treatment of HR (+/−) HER2-low mBC. The average time to fill the survey was around 15 minutes. Data from the survey were retrieved in an excel sheet; all experts responded to the survey, with the difference that the specific treatment questions did not apply to the pathology experts. No formal statistical analysis was used. The responses grouped under the main topics were further discussed in detail during the meeting, while experts agreed that the structure of the manuscript will follow these topics. For each of these, the thought leaders discussed the institutional or national data versus literature, described the unmet needs across countries, and shared their independent views and experience. Relevant data discussed in the medical community with regard to the spectrum of the HER2-low in breast cancer were considered to firstly describe the general context and then, to a greater extent, elaborate on the local circumstances (no formal literature review). Experts identified local and/or regional challenges and constraints of clinical oncology and pathology daily practice and proposed action plans aimed at improving testing and access to treatment and, consequently, outcomes of HER2-low mBC for patients at the country and CEE level.

## Results and discussion

### Epidemiology of HER2-low breast cancer

#### Current status and challenges

The four CEE countries represented in this paper differ in terms of total population; however, in all four of these countries, BC ranks second or third in prevalence among all types of cancers and is one of the leading causes of death in women (Table 1).^25^ BC incidence rates remain high in the region and are predicted to increase in the future due to the global trend of an increasingly aging population.^26,27^ Early detection (eg screening programs) of BC is problematic in countries that have undergone economic transitions like those in CEE; many still lack clear policies and sustained investments in their medical healthcare systems.^28^ Even so, the mortality-to-incidence ratio (MIR), which is an indicator of healthcare quality, with low values indicating better care (prevention, treatment, and overall management), varies slightly and is similar to the average European value (0.27) in Slovenia and Croatia.^29^ As compared with data from 201228, we observe decreases in the MIR in all four countries, which might show that advances in cancer care have been made to some extent in the last two decades. Despite these encouraging signs, recent data show trends of increase in BC mortality in Bulgaria and Croatia in women over 45 years.^30^ In most Eastern European countries, patients with BC have a shorter OS following diagnosis compared with the rest of Europe^31^; however, in Slovenia, survival has been shown to be increasing over time.^32^ Multiple challenges and gaps in receiving optimal cancer care by individuals with mBC have been described, especially in underserved patient populations from the CEE region where socioeconomic inequalities and educational or cultural status have a considerable impact on the quality of healthcare.^33^

**TABLE 1.** Overview of cancer epidemiology across four CEE countries in 2020 (data extracted from GLOBOCAN 202025 and the European Cancer Information System^34^)

**Characteristics**	**Bulgaria**	**Croatia**	**Serbia**	**Slovenia**
Total population	6 948 445	4 105 268	8 737 370	2 078 932
Number of new cancer cases (all cancer sites)	36 451	26 092	49 043	14 180
Incidence age-standardized rate per 100 000	100	120.3	145.3	121.2
Number of new BC cases in 2020, both sexes, all ages	4061	2894	6724^a^	1410
BC new cases – rank across all types of cancers	3	2	2	3
5-year prevalence, all ages (per 100 000)	425.45	523.4	549.32	560.03
Mortality age-standardized rate per 100 000	36.3	32.8	50.9	32.3
Number of BC deaths	1533	832	2342	405
BC deaths – rank across all types of cancers	3	3	2	5
Mortality-to-incidence ratio^b^	0.36	0.27	0.35	0.27

Compared with reported rates for HER2-low cases, which range between 45% and 65% in HR+ tumors and 23% to 40% in HR-tumors^6,16,35^, local reports indicate a similar or slightly lower percentage of HER2-low cases. In a sample of 11 234 cases from the Oncology Institute of Ljubljana (Slovenia), collected from 2011 to 2021, HER2-low (1+/2+ non-amplified) was identified in 52.8% of cases. The rate of HER2 IHC 0 decreased in the last 2 years of follow-up (2020, 2021), whereas the rate of HER2-low increased.^36,37^ In Croatia, according to the National Pathohistological Breast Registry of newly diagnosed BC patients, in a sample of 8488 patients (early-stage, locally advanced, or meta-static BC), the HER2-low rate in the past 3 years was 42% (44% HER2-low in luminal A cancers, 54% in luminal B, and 36% in triple-negative BC) (unpublished data). In Serbia, in a sample of 500 patients from the Novi Sad registry, HER2-low status was identified in 50% of cases, irrespective of stage, whereas in mBC patients with testing performed only in primary tumors, the rate of HER2-low was 30% (unpublished data). For Bulgaria, no official data are available.

#### Unmet needs

Robust, more standardized data on incidence, prevalence, and mortality rates by type and stage of BC, and outcomes in specific groups that are usually underserved (i.e., men, patients with comorbidities, patients of cultural/racial/religious diversity) are scarce in the region and, consequently, very much needed. The difference between data from Western countries and those in the CEE region may be partly explained by lack of properly founded national cancer registries and clinical databases collecting systemized oncology data and, of course, variations of the healthcare systems in CEE. Progress has been made recently (for example, in January 2023 Slovenia opened the Clinical Breast Cancer Registry), and more changes are expected in the future.

#### Action plan

We outlined the following top priorities:

(1) to extract retrospective data from healthcare records in a centralized way in each country and use them as a benchmark for future studies;
(2) to expand existing registries/protocols to include all HER2-low BC patients.

These actions would more sufficiently explore the variability across countries and adequately inform diagnosis and management strategies for improving patient care in CEE countries based on recent and reliable real-world evidence. Most importantly, this would aid communication with health authorities to expedite access to effective anticancer drugs for patients in this region. Continuous monitoring and reporting of management of patients with BC on a national and potentially regional or, even better, European level, is necessary to inform healthcare policies and reforms. Exposing the weaknesses of general health-care and/or oncology systems will help to improve outcomes by addressing similar issues.

### Biology of HER2-low breast cancer

#### Current status and challenges

Whether HER2-low is a distinct biological entity or not is one of the key questions in the field of HER2-low biology.^16,38^ While the spectrum of HER2 positivity expands, no robust evidence exists to consider HER2-low a clinically distinctive entity or a definite subtype^14,39,40^, which has led some groups to conclude that such categorization remains to be clarified in the future.^17,20,41^ At a local level, a recent report from Serbia including patients with early BC has shown a higher proportion of pathologic complete response after neoadjuvant chemo-therapy in patients with HER2 IHC 0 as compared with HER2-low, indicating that new and improved treatment modalities are required for HER2-low patients.^42^

Tumor heterogeneity and tumor plasticity that traditionally characterize breast carcinomas also apply to HER2-expressing tumors.^5,43^ HER2 intratumoral (spatial) heterogeneity is a well-known phenomenon reported in up to 40% of BC.^44,45^ HER2-low expression was shown to be highly unstable during disease evolution, with a higher proportion of HER2-low rates in recurrent BC samples (temporal heterogeneity).^38,46,47^

Other matters of debate in the literature discussed were whether the efficacy of HER2-targeted treatments is higher in tumors with higher HER2 expression levels^48,49^, and how HER2-low could be an escape mechanism displayed by tumors in case of HR+-directed treatments, leading researchers to believe that most cases of mBC will become HER2-low under the pressure of endocrine therapies.^50,51,52^

#### Unmet needs

Re-biopsy availability is related to our understanding of HER2-low biology. Yet, there is no specific strategy for re-biopsy at recurrence at the country or regional level, and computed tomography (CT)-guided biopsies are difficult to access and generally rarely performed. While financing for re-biopsies may not be an issue, Serbia, for example, is hindered by a scarcity of experts who can perform biopsies, such as interventional radiologists or pulmonologists.

#### Action plan

To optimize the management and subsequently the outcomes of HER2-low BC patients in future and to address spatial and temporal tumor heterogeneity, we proposed to:

(1) Foster HER2-low early diagnosis by developing and implementing local pathways for mBC patients, with mandatory checks of previous pathology reports;
(2) perform multiple rounds of re-biopsy at each relapse of locoregional or distant metastases;
(3) keep clinicians informed of new treatment options available in their countries, based on a possible different result of the re-biopsy compared with the primary tumor biopsy.

### Pathologic diagnosis of HER2-low breast cancer

#### Current status and challenges

Historically, HER2 expression was classified in a binary way: positive or negative.^44,53^ New evidence indicates that patients with low HER2 expression (IHC 1+ or 2+ and ISH-negative) represent a new targetable category of BC.^3^ In light of these changes, HER2 testing and reporting has become more complex.^54^

The 2023 updated guidelines issued by the American Society of Clinical Oncology (ASCO)/College of American Pathologists (CAP) for HER2 testing include no changes in prior (2018) terminology or traditional terminology of positive/equivocal/negative for HER2 IHC results but calls to increased awareness for IHC 1+ or 2+ non-amplified cases that deem patients eligible for treatment with T-DXd.^7,53^ While pathology groups state that HER2-low is a qualitative term^55^, medical oncologists use conflicting terminology for interpreting ASCO/CAP guidelines (i.e., HER2-0 with potential future categories HER2-null and HER2-ultralow, HER2-low, HER2-positive).^5^ Pathology experts agreed that HER2-low is rather an operational term, with ASCO/CAP guidelines being currently followed in pathology clinical practice. No HER2-low term is currently included in reports; however, the term HER2-negative is recommended to be changed in “HER2-negative for protein overexpression/gene amplification” since non-overexpressed levels of the HER2 protein may be present in these cases.

Another challenge is related to the companion diagnostic tests for evaluating 1+ and 2+/ISH-negative disease.^3^ Assays used are either those approved and currently available on the market (with Ventana HER2/neu 4B5 [F. Hoffmann-La Roche Ltd] being more frequently used in the CEE area) or ones developed in-house. Besides temporal and spatial tumor heterogeneity, many other factors are known to impact the IHC scoring – from pre- and post-analytical factors to test sensitivity, type of specimen, and laboratory and/or reader experience.^3,6,56,57,58^

Among the specific pathology challenges mentioned at local level, the following were underlined: in Bulgaria, lack of reimbursement for ISH (ISH tests are paid for by patients), lack of continuous medical education for pathologists to train on the changing paradigm of HER2 assessment, reporting and its relevance to treatment, lack of certification process of either pathology laboratories or clinical centers, and no quality control processes in place. In Serbia, previous discordance in IHC detection of HER2 between national pathology laboratories was reported (the overall agreement ranged between 79% and 89%), with discrepancies on chromogenic ISH indicating a misdiagnosis rate of almost 16%.^59^ In Croatia, in a sample of 126 patients, discordance in HER2 scoring between central and local laboratories was 12% – results that are in line with the literature.^60,61^ The sources of error in the local study were partly pre-analytical and partly analytical, thus emphasizing the need for rigorous application of standardized staining and scoring procedures for precise determination of HER2 protein level, which is particularly important in the HER2-low group. The experts from Bulgaria added that a high variability of HER2 testing results between pathology centers also applies in their country, leading to high number of retesting and second opinions.

#### Unmet needs

In terms of pathology diagnosis, the unmet needs identified in the four countries from the CEE region are broad and at multiple levels: specific medical education for pathologists, reimbursement of ISH testing in all countries, improved robustness of HER2 testing with current available techniques, standardization of and quality-controlled HER2 testing between centers, precise and accurate reporting systems, more homogenous inter-institutional procedures, and more certified laboratories.

#### Action plan

The action plan discussed included the following proposals:

(1) to improve pre-analytical and analytical phases of HER2 testing and to reduce false-negative/false-positive reports, a rigorous internal and external quality control is required at every institutional level;
(2) to increase awareness of HER2-low testing and scoring and to improve reporting, virtual meetings, and live workshops for pathology specialists, as well as multidisciplinary meetings of all specialists involved in the management of HER2-low BC patients, should be formally organized in each country;
(3) to improve HER2-low score accuracy and reduce inter-laboratory variability, participation in ring studies is highly encouraged;
(4) to increase comparability across various geographies and build best practices, center-, country-, and regional-level monitoring and reporting of pathology results is recommended.

### Treatment of HER2-low breast cancer

#### Current status and challenges

CEE countries are characterized by a variable reimbursement status of anticancer drugs for HER2-negative mBC (Table 2). In Slovenia the majority of treatments are reimbursed, irrespective of line of treatment; however, T-DXd is not yet reimbursed for HER2-low BC. In Croatia, despite innovative treatments being reimbursed, their use in later line (third line [3L]+) depends on budgetary decisions at the institutional level, potentially introducing disparity in the treatment of mBC patients in need. By contrast, in Bulgaria, reimbursement is granted in general in any line for all drugs approved at the European level for HER2-positive mBC, which facilitates treatment sequencing; however, this does not apply for HER2-low mBC. Serbia faces the biggest challenges in the region, with innovative drugs being available for first-line (1L) and second-line (2L)/3L HER2-positive mBC, while treatment options for metastatic triple-negative and HR+ BC are being limited. For example, only cyclin-dependant kinase 4 and 6 (CDK4/6) inhibitors as innovative medicines are being reimbursed in 1L and 2L for HR+ mBC. By contrast, alpelisib, poly(adenosine diphosphate ribose) polymerase (PARP) inhibitors, and the ADCs sacituzumab govitecan and T-DXd can be approved in some specific circumstances, but only in later lines when other therapy options are exhausted. In consequence, the meaningful clinical applicability of the drug is significantly decreased, because rates of treatment success in later lines are rather small.

**TABLE 2.** Status of reimbursement for anticancer drugs used for treatment of HER2-negative mBC in Bulgaria, Croatia, Serbia, and Slovenia, including the year of reimbursement of at least one representative of the class

**Treatment**	**Bulgaria^a^**	**Croatia**	**Serbia^c^**	**Slovenia^d^**
CDK4/6 inhibitors	2018	2018	2022	2018
Alpelisib	2023	2021	2023	2021
PARP inhibitors	2023	2022	2021	2021
Sacituzumab govitecan			2023	2022
Trastuzumab deruxtecan			2022	2023
Atezolizumab nab-paclitaxel		2022	2021	2020
Pembrolizumab	2023^b^		2022	2023
Everolimus	By >10 years		2021	2010
Fulvestrant	By >10 years	By >10 years	2019	2004
Aromatase inhibitors	By >10 years	By >10 years	2008	By >20 years

Green = reimbursed; orange = not reimbursed, but available through early access programs or out-of-pocket expenses; red = not reimbursed; CDK4/6 = cyclin-dependent kinase 4 and 6; HER2 = human epidermal growth factor receptor 2; mBC = metastatic breast cancer; PARP = poly(adenosine diphosphate ribose) polymerase;

a In Bulgaria, PARP inhibitors are available in early breast cancer and *BRCA*-positive tumors after lack of complete response in the neoadjuvant setting. In the metastatic setting, PARP inhibitors have been reimbursed since 2019, everolimus is reimbursed in metastatic estrogen receptor-positive (ER+) BC, and aromatase inhibitors are reimbursed in ER+ BC;

b In Bulgaria, pembrolizumab is reimbursed only in triple-negative BC within HER2-negative BC;

c In Serbia, medications in orange are registered and could be used in special circumstances but are not reimbursed/covered by public health insurance for HER2-negative mBC;

d In Slovenia, sacituzumab govitecan is reimbursed for triple-negative BC only, and trastuzumab deruxtecan for HER2-positive BC only.

In all four CEE countries, the ESMO guidelines and, in some countries, local guidelines with applicable updates are followed.^62,63,64^ Whereas treatment decisions in 1L are aligned across countries, experts agreed that decisions in 2L/3L are individualized based on patient and tumor characteristics and treatment outcomes, although these decisions are highly dependent on reimbursement conditions. While innovative treatments are usually approved in Europe through a centralized procedure via the European Medicines Agency^65^, the high costs of new anticancer drugs restrict their use until reimbursement. Previous reports from CEE have shown significant delays from marketing authorization to reimbursement of novel oncology medicines and reduced numbers of available drugs in this region, which undoubtedly leads to worsening patient outcomes.^21,24,66^ For example, perhaps due to diverse reimbursement models and policies and lack of sustained investment in the oncology field, trastuzumab, one of the essential medicines for treatment of HER2-positive BC, did not receive full reimbursement in Eastern Europe and, with the exception of Slovenia and Croatia, was insufficiently procured to allow treatment access to all patients in need for several years.^22,23,28^

We have identified the following challenges applicable, to various extents, in all participating countries: significant delays in reimbursement decisions; limitative, restrictive reimbursement conditions that impact sequencing and/or treatment rechallenges; limited access to clinical trials with novel cancer medicines; and early access/bridging programs until treatment reimbursement. In addition, optimal sequencing remains to be determined, as levels of evidence are variable for different treatments.

#### Unmet needs

Unmet needs of equal importance for adequate access to treatment of HER2-low mBC were availability and/or full reimbursement of treatments in all lines, extension of reimbursement criteria to all lines of treatment for medicines with marketing authorization granted, and reduced times from product marketing authorization to reimbursement for novel, innovative anticancer drugs. Avoiding repeating the situation of trastuzumab reimbursement and ensuring active involvement of all stakeholders in cancer care are prerequisites for preventing disparities in treatment of HER2-low BC patients between CEE countries.

#### Action plan

**FIGURE 1.**
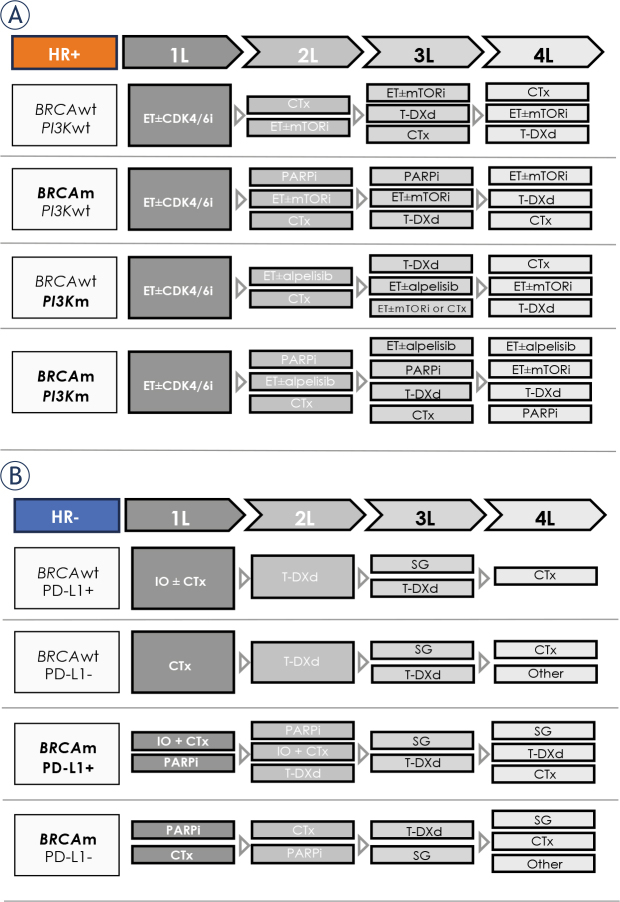
Algorithms for HER2-low mBC in light of evolving treatment paradigms, according to the HR status and other actionable targets: **(A)** HR+ and **(B)** HR−. The ideal scenario considers availability of all treatments in all lines and unrestricted treatment access. 1L/2L/3L/4L = first/second/third/fourth line; BRCAm = BReast CAncer gene mutations; BRCAwt = BReast CAncer gene wild type; CDK4/6i = cyclin-dependent kinase 4/6 inhibitor; CTx = chemotherapy; ET = endocrine therapy; HER2 = human epidermal growth factor receptor 2; HR = hormone receptor; IO = immunotherapy; mBC = metastatic breast cancer; mTORi = mammalian target of rapamycin inhibitor; PARPi = poly(adenosine diphosphate ribose) polymerase inhibitor; PD-L1 = programmed death-ligand 1; PI3Km = phosphatidylinositol 3-kinases mutations; PIK3wt = phosphatidylinositol 3-kinases wild type; SG = sacituzumab govitecan; T-DXd = trastuzumab deruxtecan. Treatment in 2L, 3L, 4L, and further lines is based on: previous therapy received; duration of response to previous treatment; patient’s preferences, condition, and comorbidities; toxicities of previous therapies; presumed benefit of further lines of therapy; and treatment availability. Per current approved label, trastuzumab deruxtecan as monotherapy is indicated for the treatment of adult patients with unresectable or metastatic HER2-low breast cancer who have received prior chemotherapy in the metastatic setting or developed disease recurrence during or within 6 months of completing adjuvant chemotherapy. Per current approved label, sacituzumab govitecan as monotherapy is indicated for the treatment of adult patients with unresectable or metastatic triple-negative BC who have received two or more prior systemic therapies, including at least one of them for advanced disease.

Key actions for optimizing access to HER2-low BC treatments are summarized below:

(1) While changing the reimbursement models at country level is beyond the scope of this initiative, the experts propose a treatment algorithm for HER2-low mBC aligned to the current evidence, guidelines, and clinical practice, according to HR status and other actionable targets, provided there is no limited access to treatments, including availability in all lines, and patients are not in visceral crisis (Figure 1).
(2) To prevent delays or lack of any patient access to treatments proven to prolong survival, a clear process mapping of the HER2-low mBC patient journey is strongly advised. Permanent communication with local decision authorities at every level and on multiple channels should be initiated by the medical community and supported by up-to-date and sound evidence of treatment benefits. For example, lobbying for alignment to ESMO-Magnitude of Clinical Benefit Scale (MCBS) for fast approval and reimbursement in the CEE area for drugs with scores of 4 and 5 would provide authorities with additional documentation and a reproducible methodology to assess the magnitude of the benefits ensured by novel anticancer drugs.^67^ In addition, involving patient organizations to advocate change policies to improve access to medicines and cancer outcomes is needed. Although the actual role played by patient representatives across each country is less known and expected to vary, their inclusion into the open dialogue with the authorities should be encouraged and supported by clinicians.

## Conclusions

This paper presents the opinions of oncology and pathology experts from four CEE countries on the optimal management of HER2-low mBC. Existing barriers to rapid diagnosis were identified, and treatment choices were proposed for real-world settings. Gaps and differences in the local epidemiology data on HER-2 low BC, certification of pathology laboratories and quality control, and availability of anticancer drugs for HER2-negative mBC across the CEE countries were identified. Preventing delays in HER2-low mBC patient access to diagnosis and timely and as-per guidelines therapies is crucial to improve outcomes.

Pathology reports should no longer report binary results as HER2-positive or -negative but include (ideally) the category of HER2-low and detail the positive score through the number of “+” because this is now becoming critical for treatment decisions. Clinicians should have pro-active discussions with policymakers and stakeholders, including patients and their representatives, in order to enable advances in HER2-low mBC diagnosis and treatment to truly optimize patient outcomes in the CEE region.

## Supplementary Material

Supplementary Material Details
