# Supplementary material for: Advancing HER2-low breast cancer management: enhancing diagnosis and treatment strategies
Source: Radiol Oncol. 2024 Jun 11;58(2):258–67. doi: 10.2478/raon-2024-0030 (PMC11165977; doi:10.2478/raon-2024-0030)

sciendo Radiology and Oncology | Ljubljana | Slovenia | www.radioloncol.com
RADIOLOGY AND ONCOLOGY

Advancing HER2-low breast cancer management: enhancing diagnosis and treatment strategies

Simona Borstnar, Ivana Bozovic-Spasojevic, Ana Cvetanovic, Natalija Dedic Plavetic, Assia Konsoulova, Erika Matos, Lazar Popovic, Savelina Popovska, Snjezana Tomic, Eduard Vrdoljak

doi: 10.2478/raon-2024-0030

HER2-Low Questionnaire

10	14:48	Active
Responses	Average time to complete	Status

1. Which country are you from?

Slovenia	2
Croatia	3
Serbia	2
Bulgaria	3

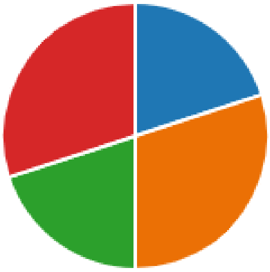

2. Are you aware of the incidence, prevalence and mortality for **HER-2 neg** patient population in your country?

Yes	3
No	2
Not sure	5

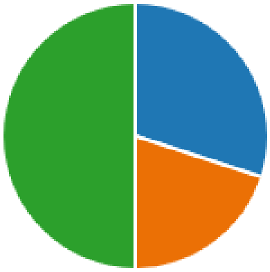

3. If *yes*, please state the incidence of HER2-neg patients in your country.

6
Responses

Latest Responses

"1230"

"3500"

"I have no information."

1 respondents (**17**%) answered **450** for this question.

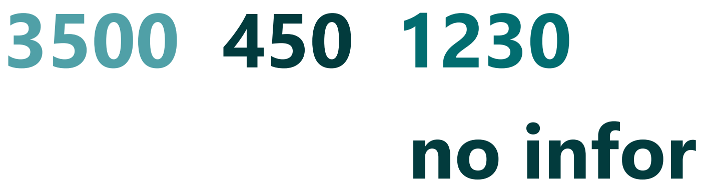

4. If *yes*, please state the prevalence of HER2-neg patients in your country.

6
Responses

Latest Responses

"16500"

"80%"

"NA"

1 respondents (**17**%) answered **16500** for this question.

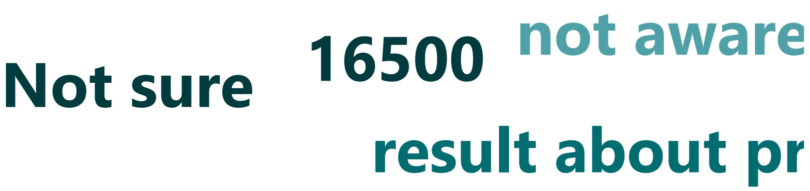

5. If *yes*, please state the mortality of HER2-neg patients in your country.

4
Responses

Latest Responses
"NA"

6. Are you aware of the incidence, prevalence and mortality for **HER-2 low** patient population in your country?

Yes	1
No	5
Not sure	4

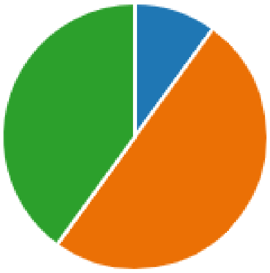

7. If *yes*, please state the incidence of HER2-low patients in your country.

3
Responses

Latest Responses
"760"
"MA"

8. If *yes*, please state the prevalence of HER2-low patients in your country.

3
Responses

Latest Responses
"8710"
"MA"

9. If *yes*, please state the mortality of HER2-low patients in your country.

3
Responses

Latest Responses
"ASR pet 100000 : 14,7"
"NA"

10. From your clinical practice, what is the percentage of HER2-low (IHC+1, IHC2+/ISH-) among advanced BC patients?

<40%	0
40%-50%	6
>50%	4

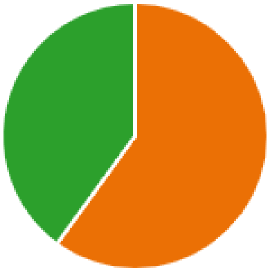

11. What do you see as the top unmet medical needs in HER2-low mBC management?

10
Responses

Latest Responses

"lack of reimbursement of T-DXd for HER2-low populatin"

"HER2 low is still and undefined as therapeutic group of patie...

"Lack of test reimbursement."

4 respondents (**40**%) answered **Reimbursement** for this question.

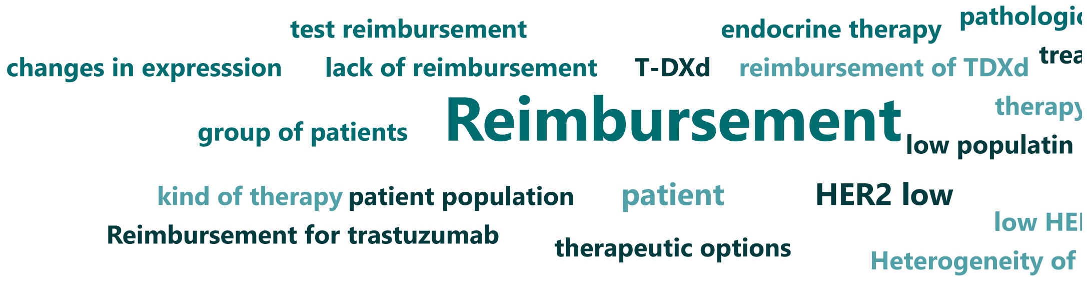

12. Do you believe that HER2-low is a targetable patient population?

Yes	10
No	0
Other	0

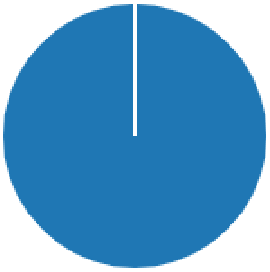

13. From your clinical practice, what is the percentage of HER2-low (IHC+1, IHC2+/ISH-) advanced BC patients who are candidates for T-DXd treatment?

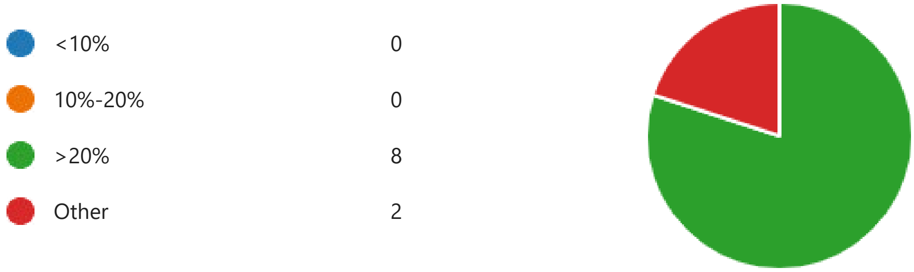

14. Would you be supportive of implementation of "HER2-low" category in pathology report instead of stating IHC+1 or IHC2+/ISH-?

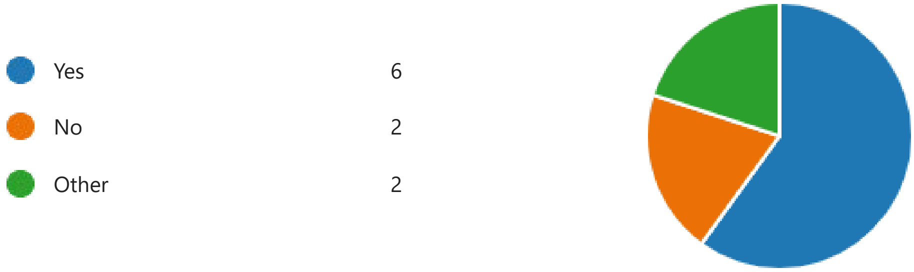

15. Which time point should be used to define a tumor as being HER2-low?

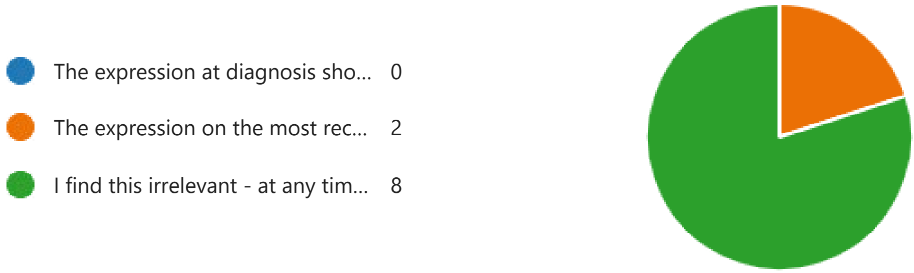

16. If all these treatment options (IO+chemo , CTx, SG, T-DXd, other) would be available in clinical practice, please list the treatment algorithm by lines of therapies for **HR -/HER2low mBC, BRCAwt, PD-L1pos**. Please use the following options, one for each line of therapy:

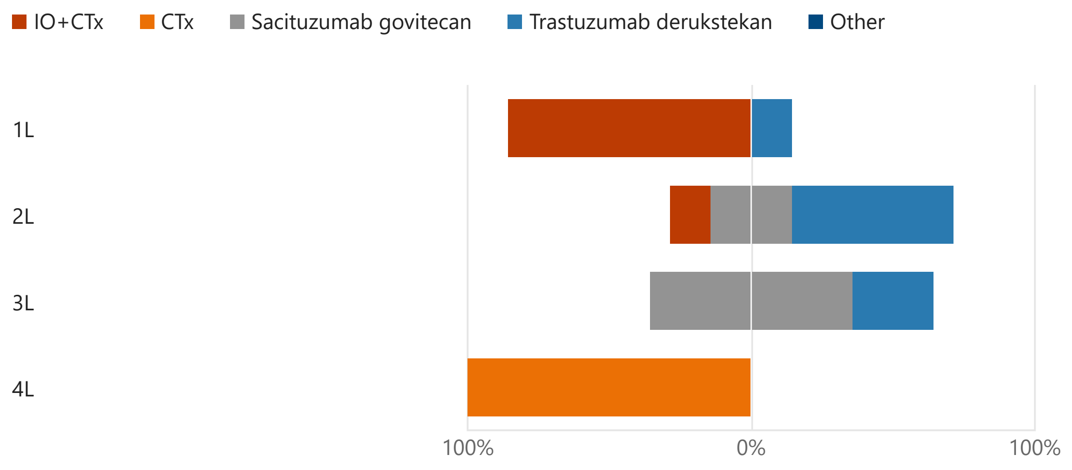

17. Please list the treatment algorithm by lines of therapies for **HR-/HER2low, BRCAwt, PD-L1pos.,** based on available treatment options in your clinical practice. Please use **ONLY** the options you have available in your clinic/country, one for each line of therapy: (IO+chemo, CTx, SG, T-DXd, other)

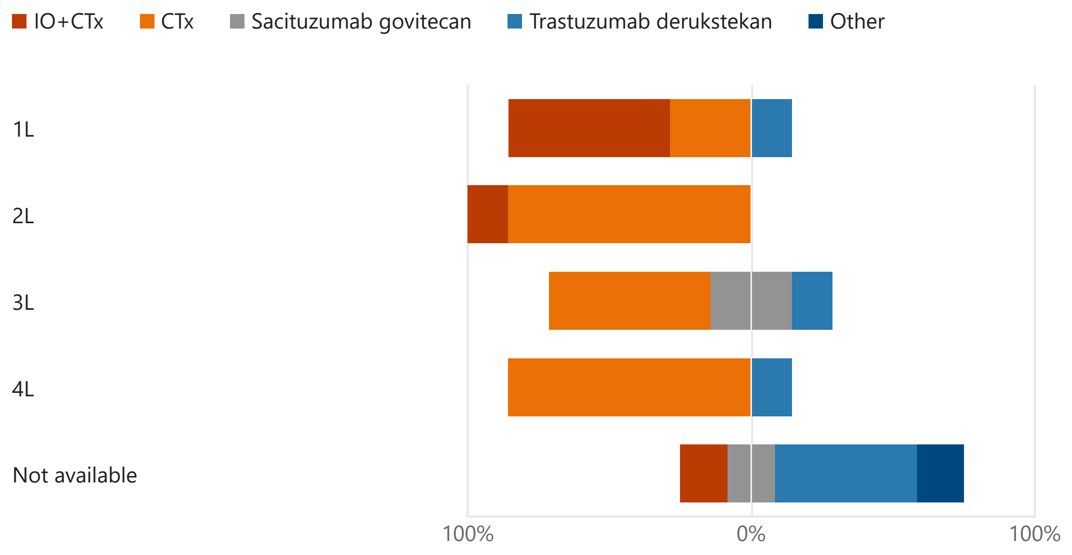

18. If all these treatment options (CTx, SG, T-DXd, other) would be available in clinical practice, please list the treatment algorithm by lines of therapies for **HR -/HER2low mBC, BRCAwt, PD-L1neg**. Please use the following options, one for each line of therapy:

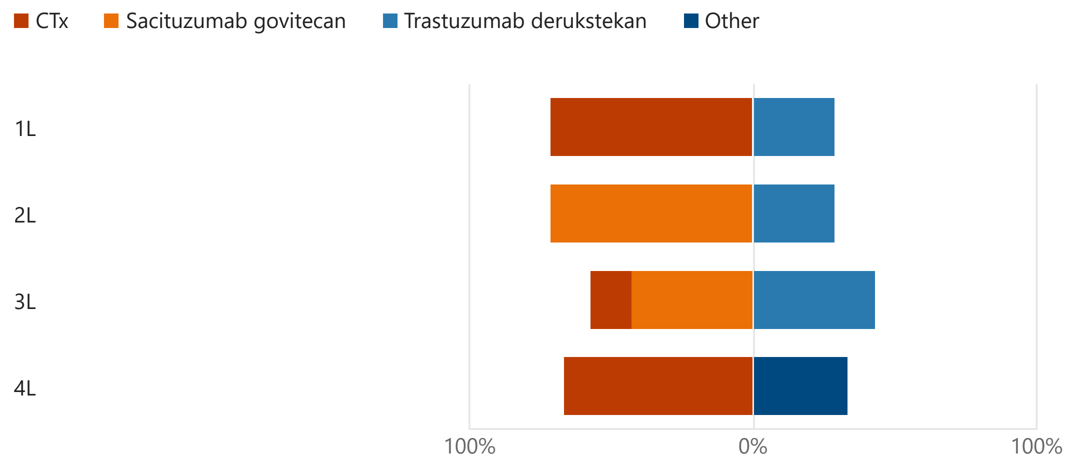

19. Please list the treatment algorithm by lines of therapies for **HR-/HER2low, BRCAwt, PD-L1neg,** based on available treatment options in your clinical practice. Please use **ONLY** the options you have available in your clinic/country, one for each line of therapy: (CTx, SG, T-DXd, other)

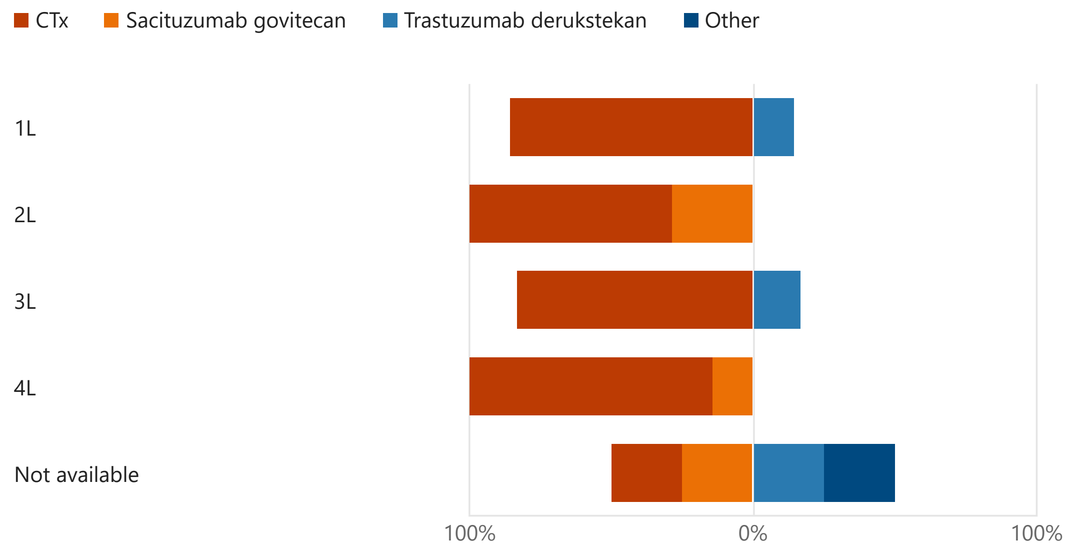

20. If all these treatment options (CTx, SG, T-DXd, PARPi other) would be available in clinical practice, please list the treatment algorithm by lines of therapies for **HR -/HER2low mBC, BRCAm, PD-L1neg**. Please use the following options, one for each line of therapy:

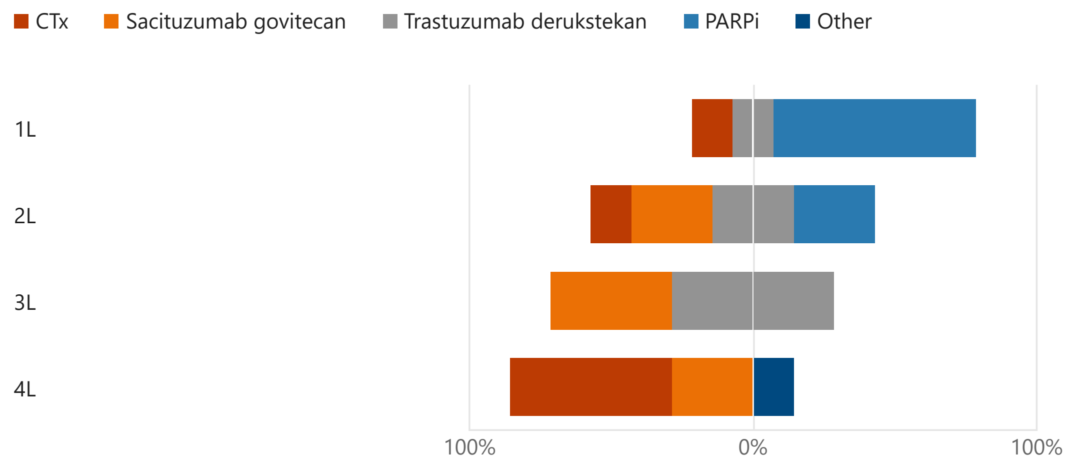

21. Please list the treatment algorithm by lines of therapies for **HR-/HER2low, BRCAm, PD-L1neg,** based on available treatment options in your clinical practice. Please use **ONLY** the options you have available in your clinic/country, one for each line of therapy: (CTx, SG, T-DXd, PARPi, other)

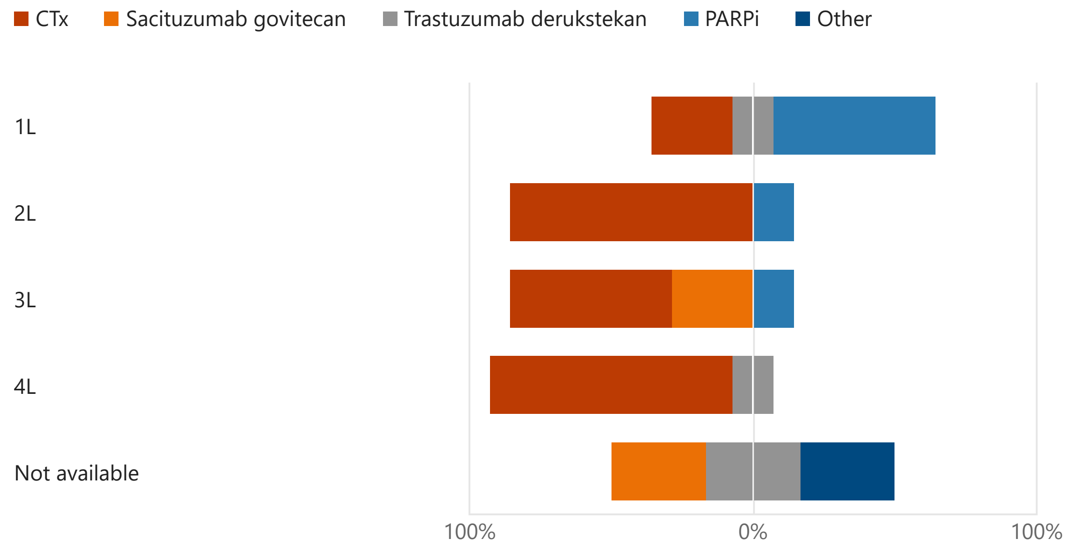

22. If all these treatment options (IO+chemo , CTx, SG, T-DXd, PARPi other) would be available in clinical practice, please list the treatment algorithm by lines of therapies for **HR -/HER2low mBC, BRCAm, PD-L1pos**. Please use the following options, one for each line of therapy:

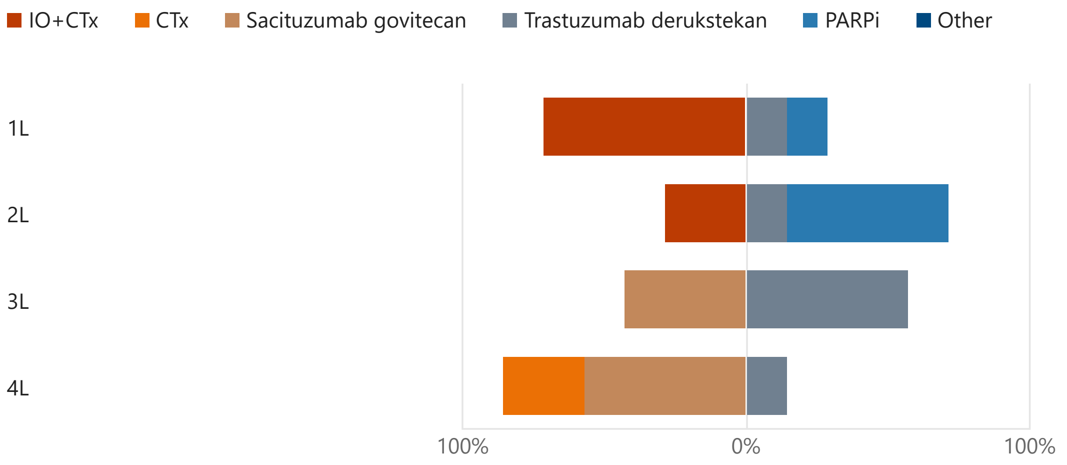

23. Please list the treatment algorithm by lines of therapies for **HR-/HER2low, BRCAm, PD-L1pos,** based on available treatment options in your clinical practice. Please use **ONLY** the options you have available in your clinic/country, one for each line of therapy: (IO+chemo, CTx, SG, T-DXd, PARPi, other)

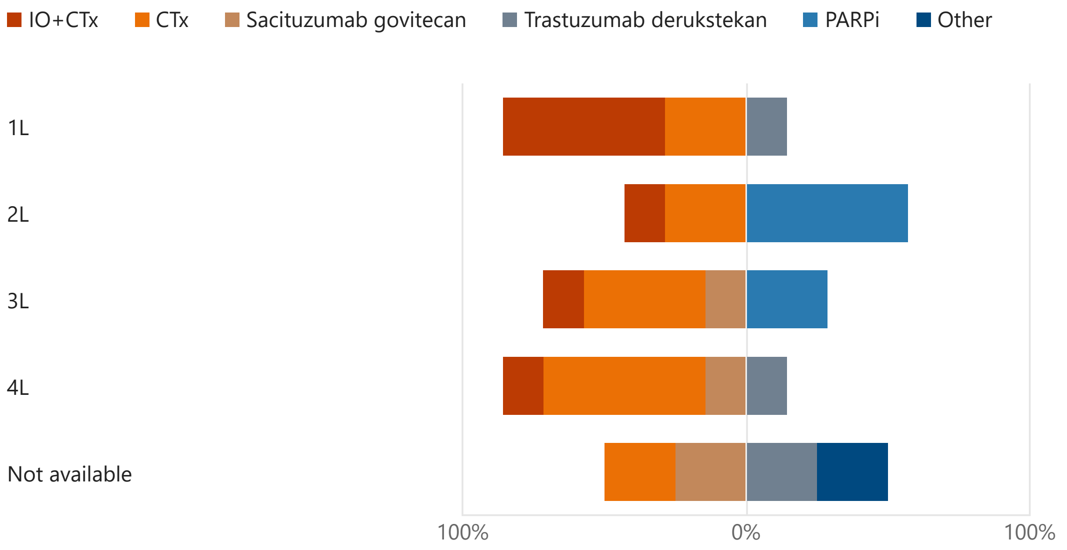

24. If all these treatment options (ET±CDK4/6i, ET±mTORi, CTx,T-DXd, other) would be available in clinical practice, please list the treatment algorithm by lines of therapies for **HR+/HER2low, BRCAwt, PI3Kwt**. Please use the following options (ET±CDK4/6i, ET±mTORi, CTx, T-DXd, Other)

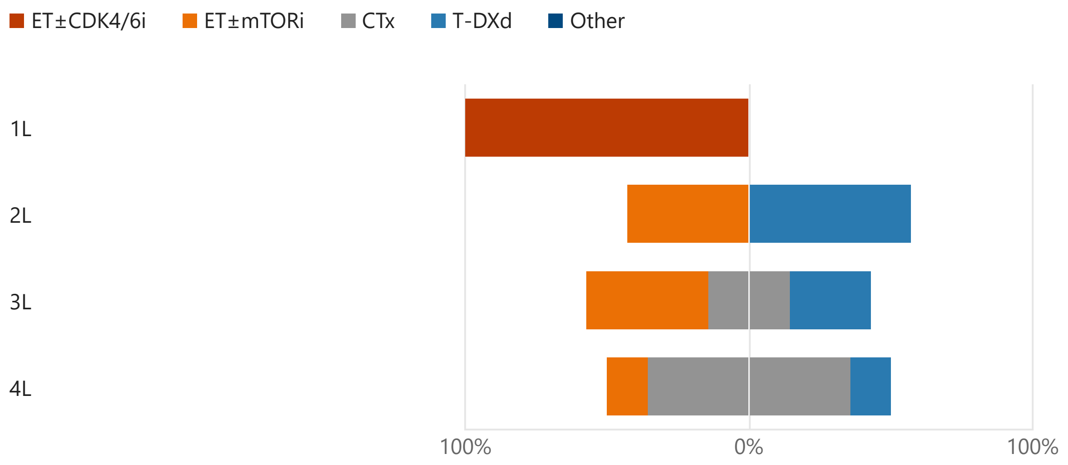

25. Please list the treatment algorithm by lines of therapies for **HR+/HER2low, BRCAwt, PI3Kwt** based on available treatment options in your clinical practice. Please use **ONLY** the options you have available in your clinic/country, one for each line of therapy: (ET±CDK4/6i, ET±mTORi, CTx, T-DXd, Other)

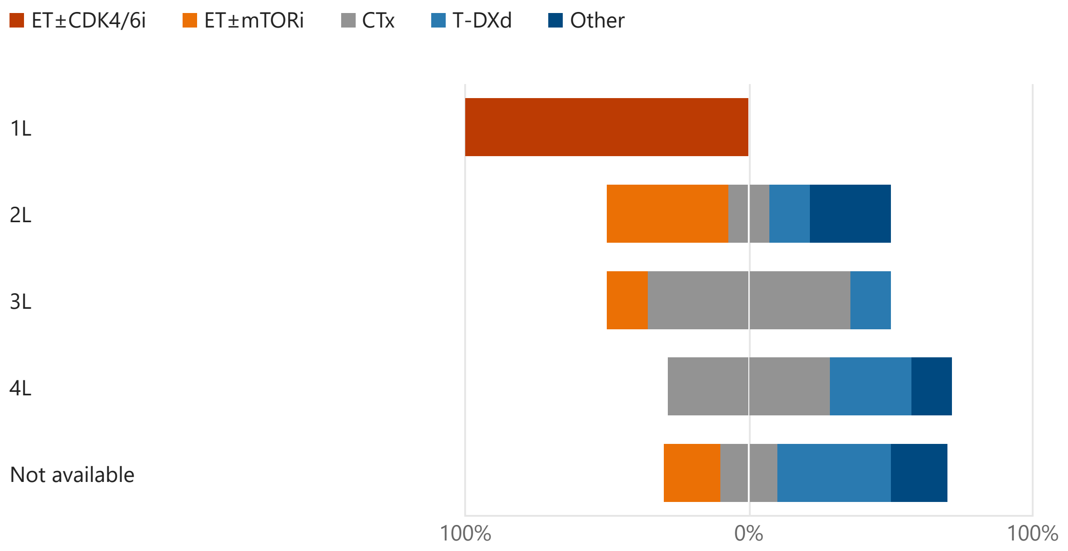

26. If all these treatment options (ET±mTORi, CTx, PARPi, T-DXd, other) would be available in clinical practice, please list the treatment algorithm by lines of therapies for **HR+/HER2low, BRCAm, PI3Kwt**. Please use the following options (ET±CDK4/6i, ET±mTORi, PARPi, CTx, T-DXd, Other)

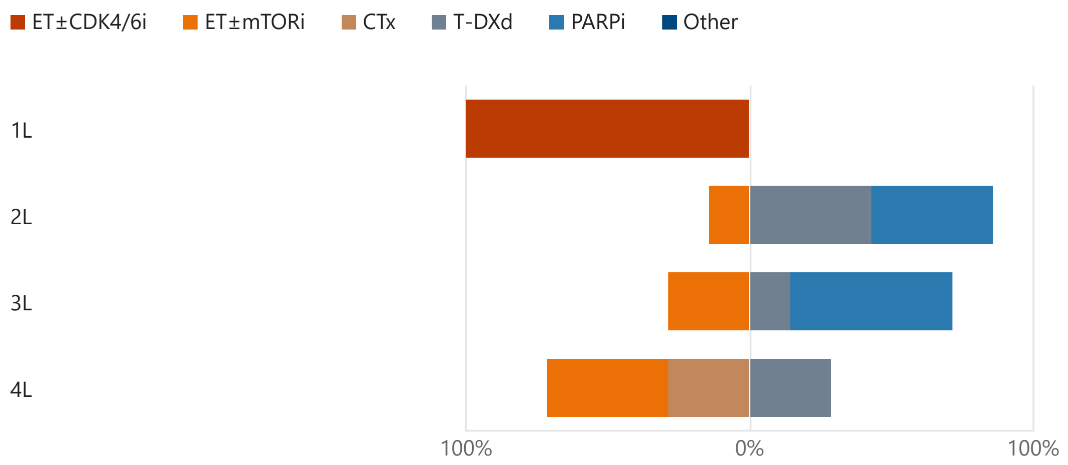

27. Please list the treatment algorithm by lines of therapies for **HR+/HER2low, BRCAm, PI3Kwt** based on available treatment options in your clinical practice. Please use **ONLY** the options you have available in your clinic/country, one for each line of therapy: (ET±CDK4/6i, ET±mTORi, PARPi, CTx, T-Dxd, Other)

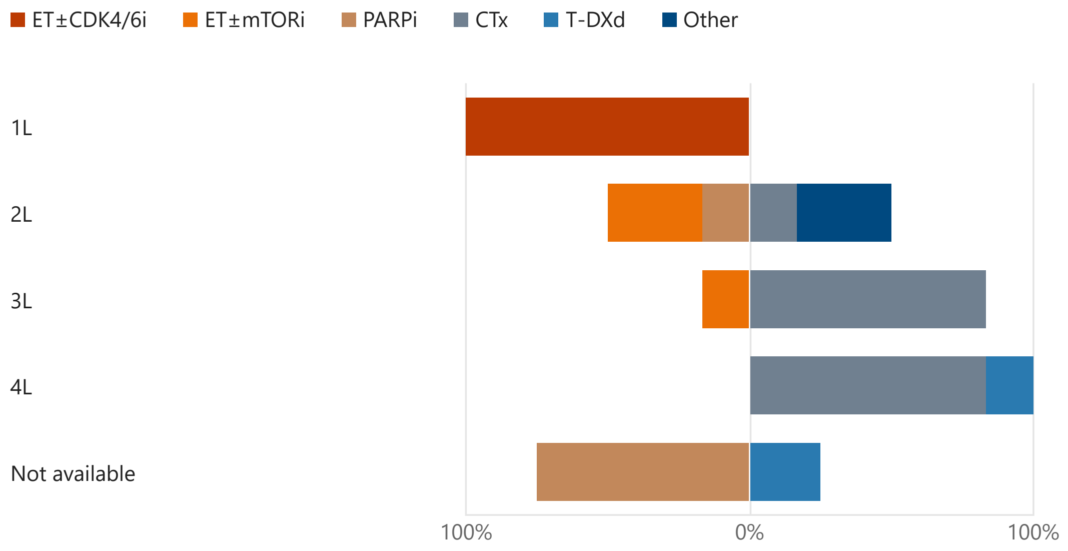

28. If all these treatment options (ET±mTORi, ET±alpelisib, CTx,T-DXd, other) would be available in clinical practice, please list the treatment algorithm by lines of therapies for **HR+/HER2low, BRCAwt, PI3Km**. Please use the following options (ET±CDK4/6i, ET±mTORi, ET±alpelisib, CTx, T-DXd, Other)

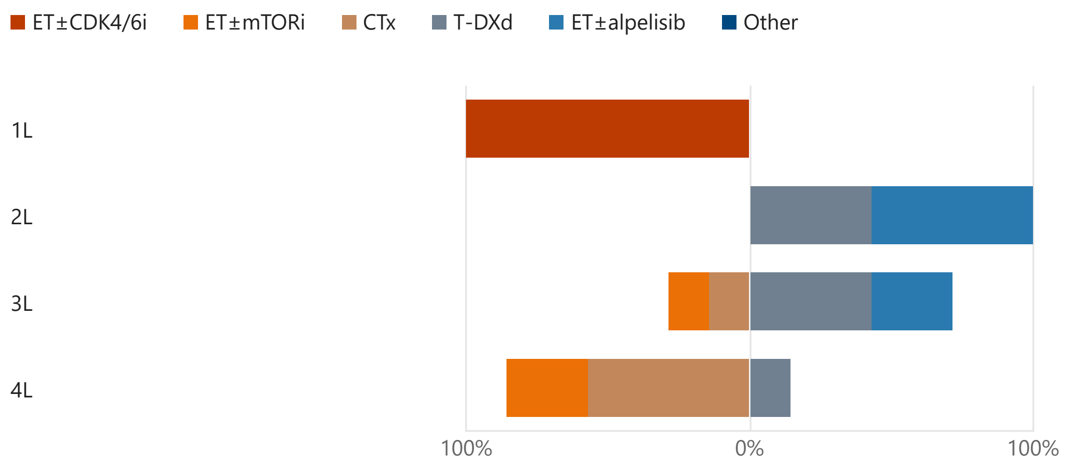

29. Please list the treatment algorithm by lines of therapies for **HR+/HER2low, BRCAwt, PI3Km** based on available treatment options in your clinical practice. Please use **ONLY** the options you have available in your clinic/country, one for each line of therapy: (ET±CDK4/6i, ET±mTORi, ET±alpelisib, CTx, T-DXd, Other)

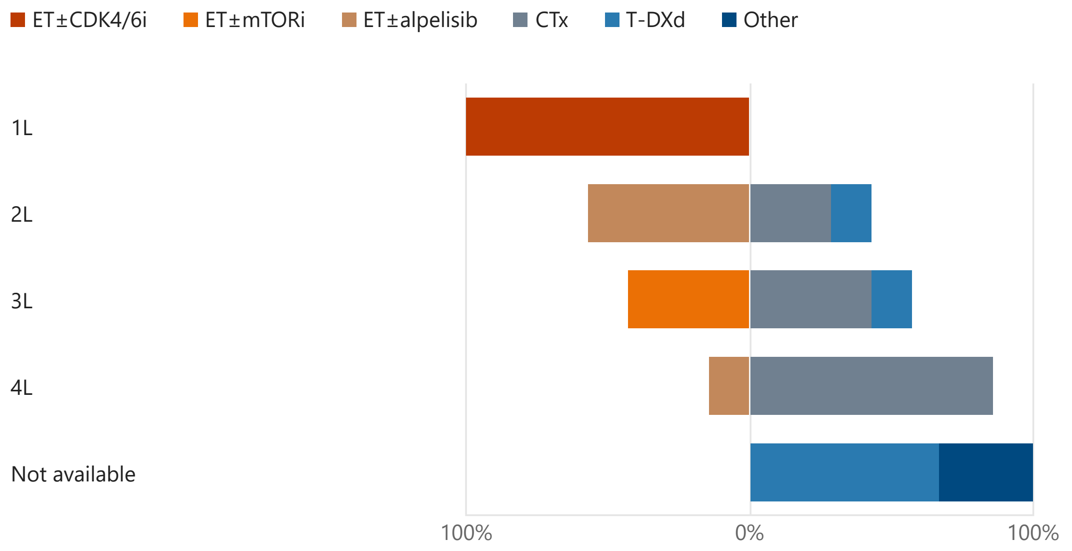

30. If all these treatment options (ET±mTORi, ET±alpelisib, CTx, PARPi, T-DXd, other) would be available in clinical practice, please list the treatment algorithm by lines of therapies for **HR+/HER2low, BRCAm, PI3Km**. Please use the following options (ET±CDK4/6i, ET±mTORi, ET±alpelisib, CTx, T-DXd, PARPi)

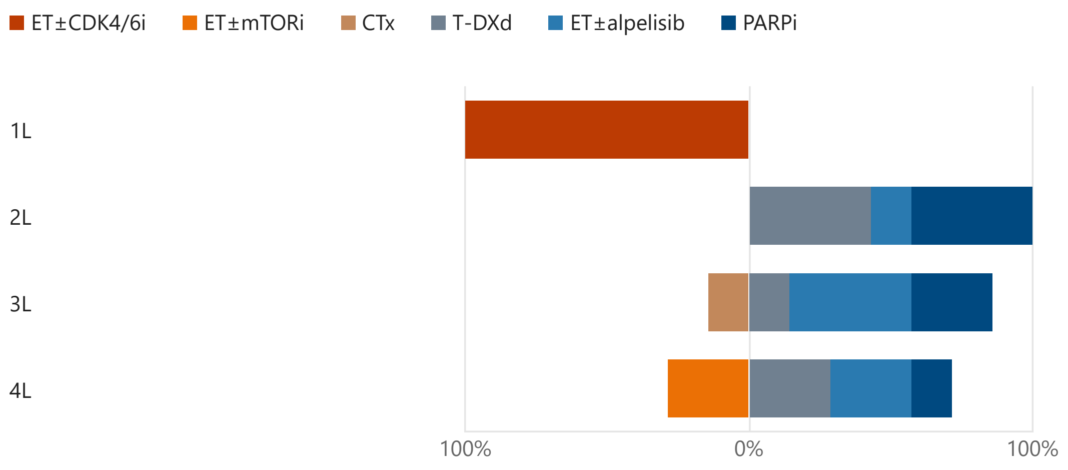

31. Please list the treatment algorithm by lines of therapies for **HR+/HER2low, BRCAm, PI3Km** based on available treatment options in your clinical practice. Please use **ONLY** the options you have available in your clinic/country, one for each line of therapy: (ET±CDK4/6i, ET±mTORi, ET±alpelisib, CTx, T-DXd, PARPi)

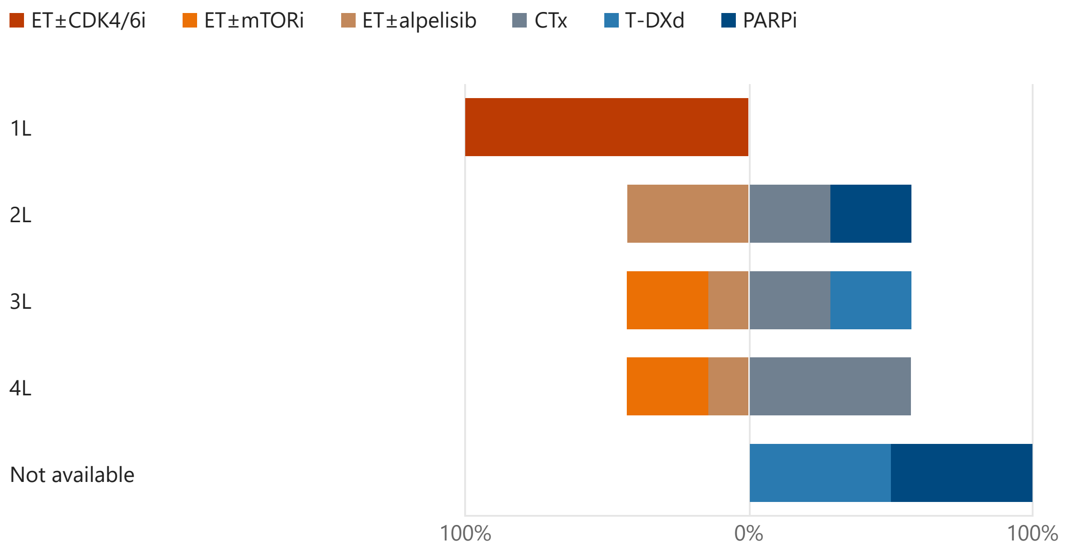

Supplement: Supplementary file 1 — Supplementary Material Details [file raon-2024-0030-sm.pdf]
